# Development of a machine learning model for predicting pediatric mortality in the early stages of intensive care unit admission

**DOI:** 10.1038/s41598-020-80474-z

**Published:** 2021-01-13

**Authors:** Bongjin Lee, Kyunghoon Kim, Hyejin Hwang, You Sun Kim, Eun Hee Chung, Jong-Seo Yoon, Hwa Jin Cho, June Dong Park

**Affiliations:** 1 Department of Emergency Medicine, Seoul National University Hospital, Seoul, Korea; 2 Department of Biomedical Engineering, Seoul National University College of Medicine, Seoul, Korea; 3 Department of Pediatrics, College of Medicine, The Catholic University of Korea, Seoul, Korea; 4 Department of Pediatrics, Chungnam National University School of Medicine, Daejeon, Korea; 5 Department of Pediatrics, Seoul National University College of Medicine, 101 Daehak-ro, Jongno-gu, Seoul, 03080 Korea; 6 Wide River Institute of Immunology, Seoul National University, Hongcheon, Korea; 7 Department of Pediatrics, Chonnam National University Children’s Hospital and Medical School, 42 Jebong-ro, Hak-dong, Dong-gu, Gwangju, South Korea

**Keywords:** Paediatric research, Machine learning

## Abstract

The aim of this study was to develop a predictive model of pediatric mortality in the early stages of intensive care unit (ICU) admission using machine learning. Patients less than 18 years old who were admitted to ICUs at four tertiary referral hospitals were enrolled. Three hospitals were designated as the derivation cohort for machine learning model development and internal validation, and the other hospital was designated as the validation cohort for external validation. We developed a random forest (RF) model that predicts pediatric mortality within 72 h of ICU admission, evaluated its performance, and compared it with the Pediatric Index of Mortality 3 (PIM 3). The area under the receiver operating characteristic curve (AUROC) of RF model was 0.942 (95% confidence interval [CI] = 0.912–0.972) in the derivation cohort and 0.906 (95% CI = 0.900–0.912) in the validation cohort. In contrast, the AUROC of PIM 3 was 0.892 (95% CI = 0.878–0.906) in the derivation cohort and 0.845 (95% CI = 0.817–0.873) in the validation cohort. The RF model in our study showed improved predictive performance in terms of both internal and external validation and was superior even when compared to PIM 3.

## Introduction

The prediction of mortality in intensive care units (ICUs) helps guide therapeutic decision-making and resource allocation. This can be applied to monitor the performance of an individual ICU and to compare the performances of different ICUs. It may also be useful in counseling family members and providing information on the prognosis of critically ill patients^1^.

Therefore, several tools have been developed to predict the mortality of critically ill pediatric patients. Among them, the Pediatric Index of Mortality 3 (PIM 3) is one of the severity scoring systems widely used with pediatric patients^2^. PIM 3 predicts the probability of mortality using specific physical signs, laboratory test results, and clinical features within one hour of admission to the ICU. It has been validated in various countries, and mortality prediction performance has been reported as the area under the receiver operating characteristic curve (AUROC), which varied between 0.75 and 0.83 depending on the center where the study was conducted^3–6^.

Meanwhile, several studies have been conducted to predict mortality with outstanding performance using a machine learning method, and predictive models with high performance given by AUROC values of 0.85–0.94 have been developed^7–9^. However, all above studies were conducted on adult patients. Since the normal range of children’s vital signs is different from that of adults, it is difficult to apply the same standards to children. A recent retrospective cohort study reported the results of predicting mortality in pediatric ICU (PICU) using deep learning technique^10^. The study showed excellent predictive performance by learning the trend of vital signs in a 24-h time window with a convolutional neural network; however, it was not suitable for predicting mortality in the early stages of ICU admission because it required a trend pattern of accumulated vital signs over the time period. Thus, we planned this study to develop and validate a machine learning model for predicting childhood mortality in the early stages of ICU admission.

## Methods

### Study setting

This retrospective cohort study was conducted at four tertiary university hospitals, and patients under the age of 18 admitted to the ICUs for the following periods at each hospital were included in the study: Seoul Saint Mary’s Hospital (from January 2010 to May 2019), Chungnam National University Hospital (from September 2011 to May 2019), Seoul National University Hospital (from July 2017 to May 2019), and Chonnam National University Hospital (from July 2017 to May 2019). All pediatric patients admitted to the ICUs, except the neonatal ICU, were included in the study. In some cases, owing to shortages of beds in PICUs or coordination with other departments, pediatric patients were also treated in adult ICUs, such as the internal medicine ICU, surgical ICU, cardiac ICU, and neurological ICU. Thus, we defined “general ICUs” for the cases in which the pediatric patients were admitted to adult ICUs rather than the PICU. Premature infants were excluded from the study based on their corrected ages.

The research was approved by the institutional review boards of each institution (approval numbers: KC18RESI0092, CNUH 2019-09-068, H-1909-006-1061, and CNUH-2019-311, respectively). All data were anonymized, the informed consent requirement was waived by the Seoul Saint Mary’s Hospital Institutional Review Board, the Chungnam National University Hospital Institutional Review Board, the Seoul National University Hospital Institutional Review Board, and the Chonnam National University Hospital Institutional Review Board. Moreover, the study was conducted in accordance with the principles of the Declaration of Helsinki.

### Data collection

Demographic data of patients, such as age and gender, ICU admission-related data (type of ICU, admission source, diagnostic categories, need for surgical or procedural intervention, status at ICU discharge, etc.), physiologic variables (blood pressure, heart rate, respiration rate, pupil reflex, etc.), laboratory test results including blood gas analysis, and clinical information such as invasive mechanical ventilation and vasoactive drug use were collected on the web-based registry, a task which was handled by one researcher in each hospital. We classified the diagnosis at ICU admission into three categories, namely, very high-, high-, and low-risk groups, the same categories used in PIM 3^2^. We focused on the most abnormal values recorded or tested within the first hour of ICU admission, starting from the time at which vital signs were initially measured after ICU admission. Individual ICU episodes for patients with multiple ICU admissions during the study period were considered independently.

### Data preprocessing

The institutions were asked to review the collected data for values that were considered to be non-physiological. The criteria for these values were heart rate (< 30 beats per minute or > 300 beats per minute), respiratory rate (< 5 breaths per minute or > 120 breaths per minute), body temperature (< 30 ℃), and oxygen saturation (< 30%). After the data collected in the registry were screened, the records meeting the above criteria were requested to be reviewed by researchers in each hospital. These researchers checked whether there was a difference between the value of each medical record and the corresponding value in the registry. By repeating this process three times, each value was checked for authenticity, and values whose authenticity were not confirmed were excluded from the analyses.

The variables were classified into categorical and continuous data. The categorical data were preprocessed by one-hot encoding, while the continuous data were further classified into two groups: an age-dependent group and an age-independent group. The variables that changed in their normal ranges with respect to age, such as blood pressure and heart rate^11–13^, were assigned to the age-dependent group, while the others were assigned to the age-independent group. For the age-dependent group, z-scores were used instead of the measured values. The z-score of each variable was derived from the age distribution of the corresponding variable in the derivation cohort, using the “generalized additive model for location, scale, and shape” and “sitar” packages in the R software^14^. For the age-independent group, each variable was standardized for feature scaling^15^. The missing value was imputed as the average value when the variable was a continuous variable; when it was a categorical variable, the missing value itself was used for analysis through one-hot encoding.

### Machine learning model development and validation

In two hospitals (i.e., Seoul National University Hospital and Chonnam National University Hospital), all children were admitted to PICUs only, and there were full-time dedicated specialists in the PICUs. In another hospital (i.e., Seoul Saint Mary’s Hospital), most children were admitted to the PICU, but some were admitted to other ICUs, and no full-time specialist was responsible for the PICU alone. In contrast, in another hospital (i.e., Chungnam National University Hospital), there was no PICU and no corresponding specialist before 2017. However, since 2017, all children have been admitted to the PICU and a full-time dedicated specialist has managed the PICU. Therefore, it was determined that Seoul National University Hospital and Chonnam National University Hospital were not suitable to be part of the validation cohort, and Chungnam National University Hospital was designated as the validation cohort, and the remaining three hospitals as the derivation cohort.

A random forest (RF) algorithm was used for machine learning, and a five-fold cross-validation method was used to evaluate the performance of the model. This method was used to separate the training dataset and the test dataset, and to prevent the machine learning model from overestimation due to a specific partitioning method. Models were developed and internally validated using this method in the derivation cohort, and these models were applied to the validation cohort for external validation.

### Outcome

The primary outcome was pediatric mortality in the early stage of ICU admission, which was defined as the period within 72 h of ICU admission. The predictive performance of the RF model developed in the derivation cohort was compared with that of PIM 3, and internal and external validations were performed in the derivation and validation cohorts, respectively. The AUROC and the area under the precision recall curve (AUPRC) were used to evaluate the predictive performance of the RF model and that of PIM 3 for pediatric mortality within 72 h of ICU admission. We compared the mean AUROC and AUPRC, as well as the 95% confidence interval (CI) of the fivefold cross-validation model, with the results of PIM 3. In the case of the RF model, the average and CI were calculated for each of the five values of area under the curve. In cases of PIM 3 in which fivefold cross-validation was not applicable, we used 1000 bootstrapping methods and calculated the CI.

### Statistical analysis

In comparing the characteristics of the two cohorts, the χ^2^ test was used for categorical variables and the Mann–Whitney U test was used for continuous variables. R version 4.0.1 (R Foundation for statistical computing, Vienna, Austria; https://www.r-project.org) was used for statistical analysis and for data preprocessing. Python version 3.6.9 (Python Software Foundation, Beaverton, OR, USA; https://www.python.org) and open libraries such as scikit-learn were used for machine learning model development^16^.

## Results

### Study population

**Figure 1:**
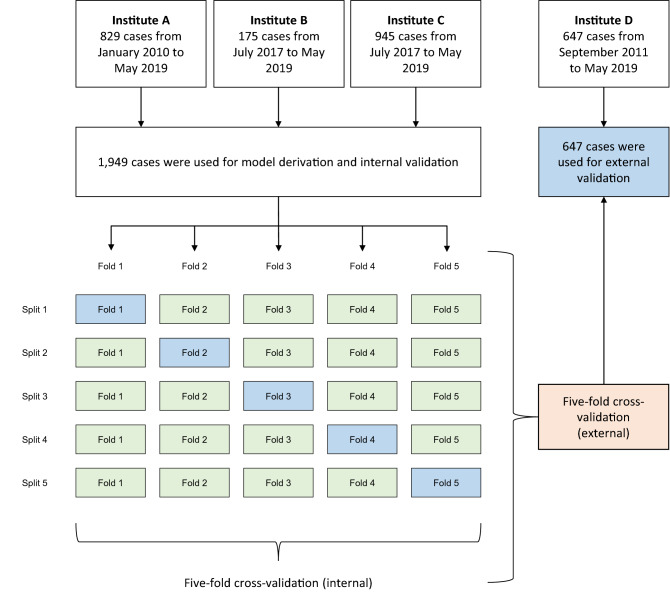
Flow chart of the study population and the internal and external validation process. Institute A: Seoul Saint Mary’s Hospital. Institute B: Seoul National University Hospital. Institute C: Chonnam National University Hospital. Institute D: Chungnam National University Hospital.

From the data collected during the study period, 1,949 cases from the derivation cohort and 647 cases from the validation cohort were used for analysis. The age (median [interquartile range]) for each cohort was 29 (4–97) months and 18 (4–111) months, respectively. Females accounted for 862 (44.2%) and 243 (37.6%) in the cohorts, respectively. Additional demographic and clinical characteristics are shown in Table 1. Figure 1 shows a flow chart of the study population and the internal and external validation process.

**Table 1:** Demographic and clinical characteristics of patients in each cohort.

Variables	Derivation cohort (*n* = 1949)	Validation cohort (*n* = 647)	*P*
Age, months	29.0 (4.0 to 97.0)	18.0 (4.0 to 111.0)	0.433^†^
Female	862 (44.2)	243 (37.6)	0.003*
Systolic blood pressure, mmHg	106.0 (91.0 to 123.0)	103.0 (88.0 to 118.0)	< 0.001^†^
Diastolic blood pressure, mmHg	56.0 (45.0 to 68.0)	61.0 (48.0 to 73.0)	< 0.001^†^
Heart rate, beats/minute	141.0 (119.0 to 162.0)	140.0 (118.0 to 158.0)	0.097^†^
Respiratory rate, breaths/minute	32.0 (25.0 to 42.0)	27.0 (22.0 to 35.0)	< 0.001^†^
Oxygen saturation, %	99.0 (95.0 to 100.0)	100.0 (97.0 to 100.0)	< 0.001^†^
pH	7.4 (7.3 to 7.4)	7.4 (7.3 to 7.4)	0.205^†^
Base excess	− 1.4 (− 4.5 to 1.5)	− 1.7 (− 5.5 to 1.8)	0.459^†^
Platelet, × 10^9^/L	221.0 (132.0 to 325.0)	236.5 (139.5 to 330.0)	0.064^†^
Potassium, mmol/L	4.1 (3.7 to 4.6)	4.1 (3.7 to 4.7)	0.001^†^
Creatinine, mg/dl	0.3 (0.2 to 0.5)	0.4 (0.2 to 0.6)	< 0.001^†^
Total bilirubin, mg/dl	0.6 (0.3 to 1.2)	0.6 (0.3 to 1.1)	0.487^†^
Prothrombin time, INR	1.2 (1.1 to 1.4)	1.3 (1.1 to 1.6)	< 0.001^†^
Activated partial thromboplastin time, second	35.2 (29.6 to 45.0)	35.9 (29.5 to 54.3)	0.061^†^
Pediatric ICU	1620 (83.1)	39 (6.0)	< 0.001*
Required invasive mechanical ventilation	1009 (51.8)	413 (63.8)	< 0.001*
Elective ICU admission	986 (50.6)	258 (39.9)	< 0.001*
Post-operation			< 0.001*
Post-operation unrelated ICU admission	1026 (52.6)	380 (58.7)	
Recovery from a bypass cardiac procedure	255 (13.1)	138 (21.3)	
Recovery from a non-bypass cardiac procedure	105 (5.4)	14 (2.2)	
Recovery from a noncardiac procedure	563 (28.9)	115 (17.8)	
Pupil reflex			0.009*
Intact	1843 (94.6)	609 (94.1)	
Fixed	106 (5.4)	38 (5.9)	
Inotropic administration	430 (22.1)	164 (25.3)	0.095*
Mortality	154 (7.9)	65 (10.0)	0.105*
Mortality within 72 h	54 (2.8)	24 (3.7)	0.281*
PIM 3 score	− 4.6 (− 5.3 to − 3.3)	− 4.2 (− 5.0 to − 3.0)	< 0.001^†^

Continuous data are presented as median (interquartile range), and categorical data as n (%).

ICU, intensive care unit; INR, international normalized ratio; PIM 3, Pediatric Index of Mortality 3.

*P-values were derived through the χ^2^ test for categorical variables.

^†^*P*-values were derived through the Mann–Whitney U test for continuous variables.

### Main outcomes

**Figure 2:**
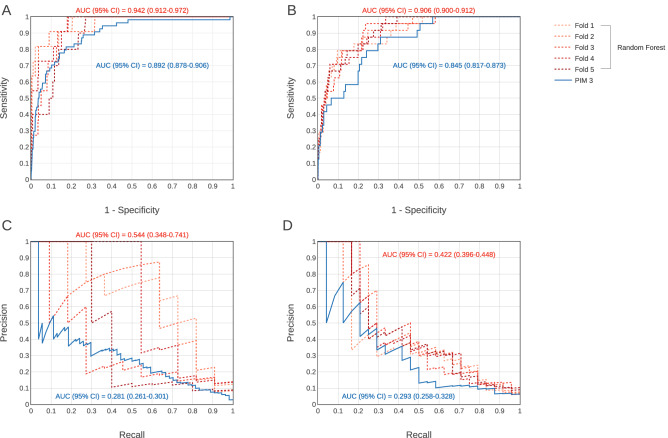
Evaluation of predictive performance of the random forest model and PIM 3. The receiver operating characteristic curves for mortality within 72 h of ICU admission from the derivation cohort are shown in (**A**), and the curves from the validation cohort are shown in (**B**). The precision-recall curve for mortality within 72 h of ICU admission from the derivation cohort are shown in (**C**), and the curves from the validation cohort are shown in (**D**). The red-based dotted curves are curves for each fold in the fivefold cross-validation process. AUC = the area under the curve, CI = confidence interval, PIM 3 = pediatric index of mortality 3.

In the case of the derivation cohort, the AUROC of the RF model was 0.942 (95% CI = 0.912–0.972) and the AUPRC was 0.544 (95% CI = 0.348–0.741). For the validation cohort, the corresponding scores were 0.906 (95% CI = 0.900–0.912) and 0.422 (95% CI = 0.396–0.448). In contrast, for PIM 3, the AUROC was 0.892 (95% CI = 0.878–0.906) and the AUPRC was 0.281 (95% CI = 0.261–0.301) for the derivation cohort, and the AUROC and AUPRC were 0.845 (95% CI = 0.817–0.873) and 0.293 (95% CI = 0.258–0.328) for the validation cohort, respectively; that is, PIM 3 showed lower predictive performance for PICU mortality compared to that of the RF model (Fig. 2). The calibration curve of the RF model is shown in Supplementary Figure S1 online.

**Figure 3:**
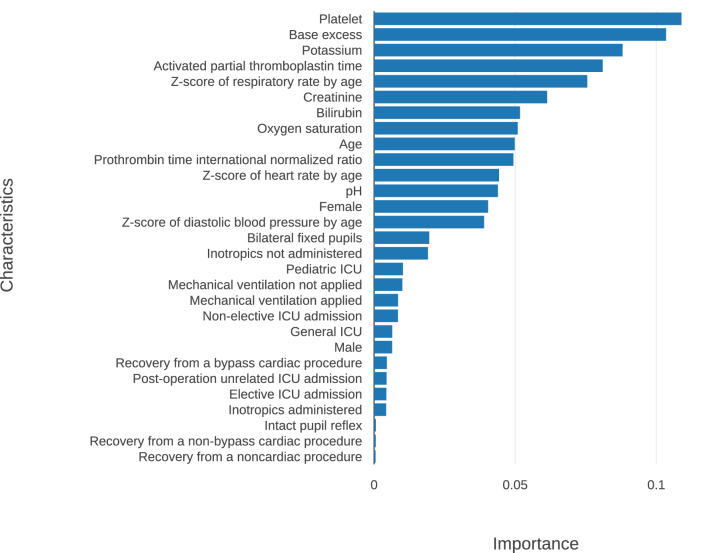
Relative importance of the random forest model variables. The importance of each feature was calculated through information gain using the difference in GINI impurity reduction. The “feature importance” function of Python’s scikit-learn library was used. ICU = intensive care unit.

The importance of variables used in the RF model learned through Gini impurity was evaluated. The variables used and their relative importance are shown in Fig. 3 with platelet, base excess, and potassium as the three most predictive values.

## Discussion

Through this study, we developed a RF model that predicts all-cause mortality for children within 72 h of ICU admission. This model was developed with the derivation cohort and validated with a validation cohort consisting entirely of different hospital patients. This is the first multicenter study comparing a clinical severity scoring system with a machine learning model to predict in-ICU mortality in children using timestamped data.

Our developed RF model showed prediction performance superior to that of PIM 3 for the following reasons. First, there was a difference in the way the age-dependent variable values were applied. In PIM 3, systolic blood pressure is used in the calculation formula without correcting for age, whereas in the RF model, the z-score according to age was used for analysis^2^. It is believed that more detailed information may have led to more precise results. Second, there was a difference in how missing values were handled. When calculating the PIM 3 score, the missing value is calculated by substituting a specific predetermined value. For example, if the systolic blood pressure is unknown, the score is calculated by assuming 120, and if the pupillary reaction to bright light is unknown, it is assumed that it is not fixed (intact). However, in our developed RF model, the missing value of the categorical variable was calculated by using the missing value itself as one variable, and the missing value of the continuous variable was calculated by substituting the average value of the variable. In other words, we worked with three categories: fixed, intact, and a separate category of ‘unknown’ where a missing value will be categorized as the latter, which is different from simply assuming unknown as intact in PIM 3. In the case of continuous variables, substituting the missing value with the average value of the variable was the same as that of PIM 3. However, we considered the differences according to age in the age-dependent variables by using age-specific z-scores. Thus, the process of missing values is more detailed than the corresponding PIM 3 method. In addition, variables not used in PIM 3 such as sex, age, and whether or not to use inotropes, were used in the RF model, which could be the reason why the RF model performance was superior.

There have been several studies on mortality prediction using machine learning. However, these studies were conducted on adult patients and were not externally validated^7–9^. Thus far, there has been only one study predicting mortality in children using machine learning. The retrospective cohort study developed a model that predicts mortality after 6–60 h of ICU admission by learning the vital signs trend of the a ‘24-h window’ in a convolutional neural network^10^. However, since it is necessary to analyze the 24-h window, it is difficult to predict results up to 24 h after ICU admission. Therefore, a limitation exists: the model using a ‘24-h window’ may not be appropriate for evaluating patients in the early stage of ICU admissions. This may mean that critically ill children who die within a few hours of ICU admission cannot be screened because of a delay in the initial use of the model. It is important to predict ICU mortality using the variables observed immediately after ICU admission, and our RF model predicts pediatric mortality within 72 h using information gathered within one hour of ICU admission.

This study has several limitations. First, the z-scores used in this study were calculated based on the distribution of the derivation cohort; thus, it may not be guaranteed that the same can be applied to other population groups. However, since it was externally verified in the validation cohort, this effect may not be very large. Second, this study only predicted mortality within 72 h of ICU admission, and no analysis was conducted to subdivide the time, such as into 6 h, 12 h, or 24 h. The 72-h period may be short, but it may also be too long; thus, there is a limitation that it cannot provide more diversified information. In addition, PIM 3, in contrast with our RF model, was developed to predict mortality during hospitalization in ICU, not mortality within 72 h of ICU admission. Thus, our model could have relative advantages over PIM 3 for mortality prediction within 72 h of ICU admission. Third, we used a web-based registry for data collection, which was contributed by one researcher in each hospital, and typographical errors could have occurred in the input process. However, we minimized human error by requesting reassessment from the researcher in each hospital up to three times, and there are measurements that can be considered non-physiologic values in the registry. Finally, data was collected over long and potentially heterogeneous periods at each hospital, but the mortality rate at each hospital has not significantly changed over time (refer to Supplementary Figure S2 online).

## Conclusions

The RF model in our study showed excellent performance in predicting pediatric mortality in the early stages (within 72 h) of ICU admission, which was demonstrated by both internal and external validation. Well-designed future studies are needed overcome the limitations of this study and further contribute to patient safety.

## Supplementary Information

Supplementary Figure S1.

Supplementary Figure S2.

Supplementary Figure Legends.

## Data Availability

The datasets generated during and/or analyzed during the current study are available from the corresponding author on reasonable request.
