# Supplementary figures and images for: Development of a machine learning model for predicting pediatric mortality in the early stages of intensive care unit admission
Source: Sci Rep. 2021 Jan 13;11:1263. doi: 10.1038/s41598-020-80474-z (PMC7806776; doi:10.1038/s41598-020-80474-z)

Fraction belonging to positive class

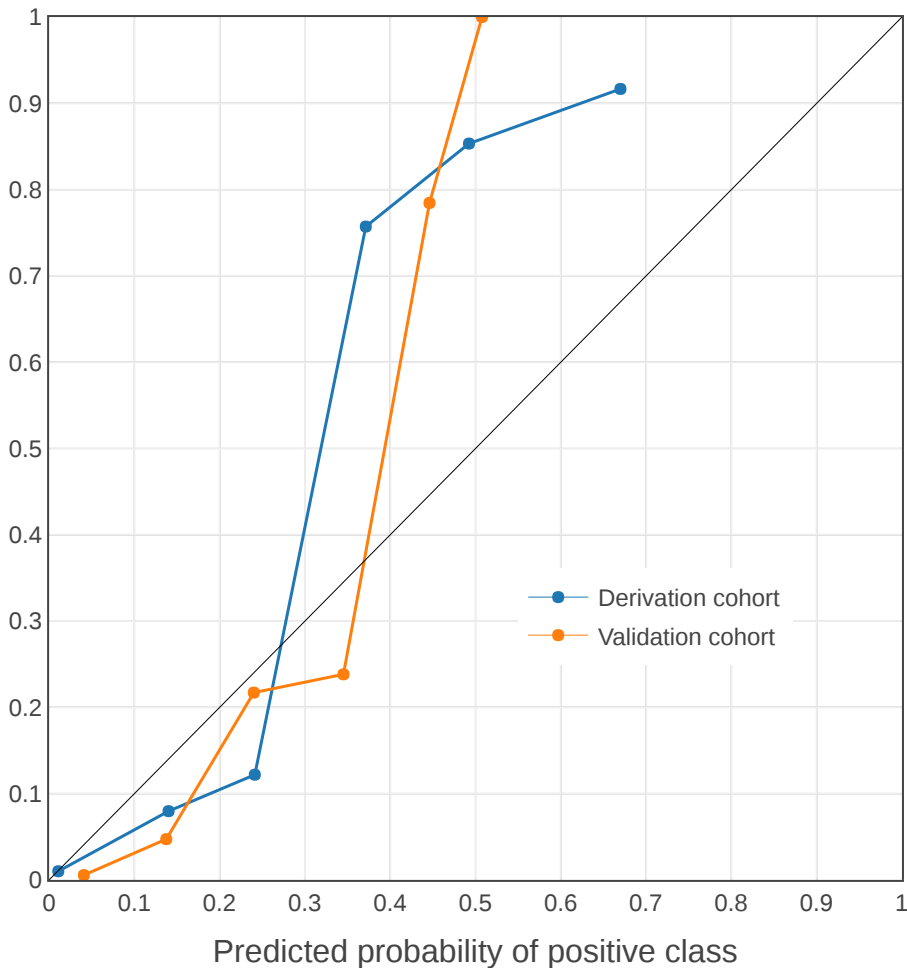

Supplement: Supplementary file 1 — Supplementary Figure S1. [file 41598_2020_80474_MOESM1_ESM.pdf]

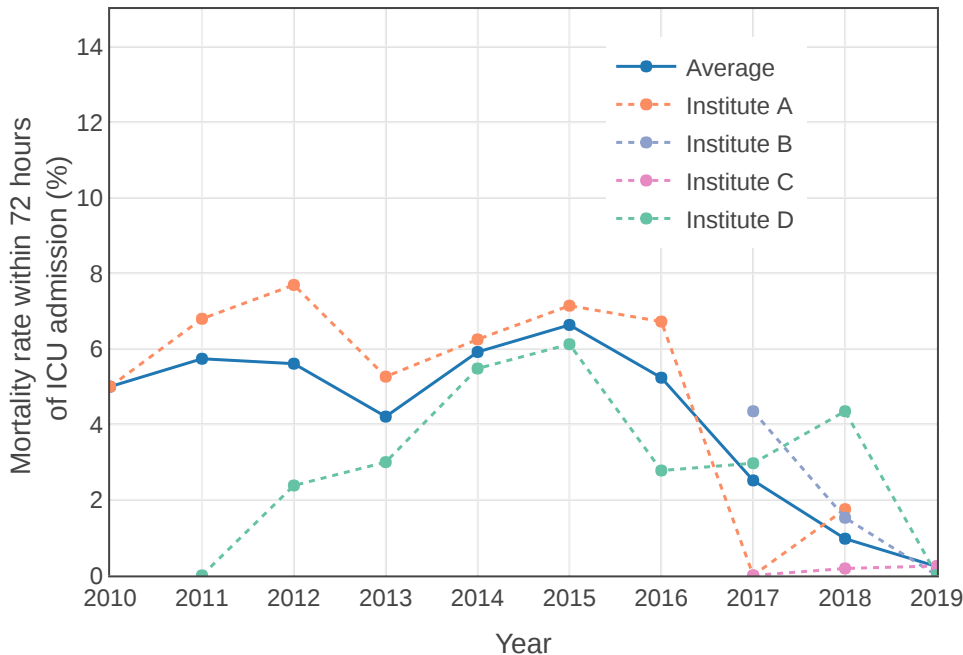

Supplement: Supplementary file 2 — Supplementary Figure S2. [file 41598_2020_80474_MOESM2_ESM.pdf]
