# Supplementary material for: Development of a machine learning model for predicting pediatric mortality in the early stages of intensive care unit admission
Source: Sci Rep. 2021 Jan 13;11:1263. doi: 10.1038/s41598-020-80474-z (PMC7806776; doi:10.1038/s41598-020-80474-z)
Supplement: Supplementary file 3 — Supplementary Figure Legends. [file 41598_2020_80474_MOESM3_ESM.docx]

**Supplementary Figure 1.** Calibration curve of the random forest model

**Supplementary Figure 2.** Linear regression analysis shows that there were no significant changes in mortality rate within 72 hours of ICU admission at each hospital over time. (Institute A *P* = 0.100, Institute B *P* = 0.108, Institute C *P* = 0.169, Institute D *P* = 0.863)

Institute A: Seoul Saint Mary’s Hospital

Institute B: Seoul National University Hospital

Institute C: Chonnam National University Hospital

Institute D: Chungnam National University Hospital

ICU = intensive care unit
